# Orbitofrontal Lesion Alters Brain Dynamics of Emotion-Attention and Emotion-Cognitive Control Interaction in Humans

**DOI:** 10.3389/fnhum.2018.00437

**Published:** 2018-11-01

**Authors:** Venla Kuusinen, Elena Cesnaite, Jari Peräkylä, Keith H. Ogawa, Kaisa M. Hartikainen

**Affiliations:** ^1^Behavioral Neurology Research Unit, Tampere University Hospital, Tampere, Finland; ^2^Faculty of Medicine and Life Sciences, University of Tampere, Tampere, Finland; ^3^Department of Neurology, Max Planck Institute for Human Cognitive and Brain Sciences, Leipzig, Germany; ^4^Department of Psychology, Saint Mary’s College of California, Moraga, CA, United States

**Keywords:** attention, cognitive control, executive function, emotion, EEG, ERP, orbitofrontal cortex, human studies

## Abstract

Patients with lesion to the orbitofrontal cortex (OFC) experience challenges in emotional control and emotion-guided behaviors. The OFC is known to participate in executive functions and attentional control of emotion and our previous research suggests OFC lesion alters the balance between voluntary and involuntary attention and cognitive control within the context of emotion. To better understand how OFC lesion affects the dynamics and interaction of these functions, we studied EEG and performance of 12 patients with lesion to the OFC and 11 control subjects with intact OFC in a Go/NoGo visual reaction time (RT) task with neutral targets and intervening threat-related emotional distractors (Executive RT Test). Event-related potentials (ERPs), specifically N2P3 peak-to-peak amplitude and the following late positive potential (LPP), were used to measure allocation of attention and cognitive control to emotional distractors. Task performance and Behavior Rating Inventory of Executive Functions—Adult version (BRIEF-A) scores were used to assess executive functions. As expected, the Control group showed increased N2P3 amplitude in the context of threat-related distractors, particularly over the right hemisphere, while LPP was not modulated by these distractors. In contrast, patients with OFC lesion showed no such impact of threat-related distractors on N2P3 amplitude but exhibited increased and prolonged left-lateralized impact of threat on LPP in the Go-condition. In NoGo-condition, the N2P3 amplitude was increased in both groups due to threat, but the impact was seen earlier, i.e., at the N2 peak in the OFC group and later at the P3 peak in Controls. The OFC group committed more errors in the Executive RT Test and reported more problems in BRIEF-A, thus both objective and subjective evidence for challenges in executive functions was obtained in patients with orbitofrontal lesion. Furthermore, the time-course of attention allocation and cognitive control towards task-irrelevant emotional stimuli was altered as evidenced by ERPs. We conclude that orbitofrontal lesion is associated with altered neural dynamics underlying the interaction of involuntary attention to emotion and cognitive control. These alterations in brain dynamics may underlie some of the challenges patients encounter in everyday life when emotional events interact with cognitive demands.

## Introduction

While little is known of human orbitofrontal cortex (OFC) function it is thought to be involved in emotional control and emotion-guided behaviors. The OFC with its wide connections to other limbic and prefrontal regions allows for integrating information of emotional value into attentional and executive function networks (Armony and Dolan, 2002; Wallis, 2007). Lesion to the OFC results in challenges in emotion-guided behaviors (Rolls and Grabenhorst, 2008) and experienced difficulties in executive functions necessary in daily life (Løvstad et al., 2012a). However, neuropsychological tests typically fail to capture any deficits in attentional, executive or affective functions in patients with lesion to OFC despite their self-reported occurrence (Manes et al., 2002; Zald and Andreotti, 2010). Alterations in emotion-attention and emotion-cognitive control interactions have been observed in these patients with event-related potentials (ERPs; Hartikainen et al., 2012a; Mäki-Marttunen et al., 2017), suggesting a lack of sensitivity on the part of traditional testing methods. More detailed knowledge, including possible changes in the dynamics of these interactions, is needed for further insight into the neural basis underlying the behavioral, emotional and cognitive challenges these patients encounter as well as for developing accurate assessment and targeted rehabilitation tools for them.

To obtain insight into the temporal dynamics of emotion-attention and emotion-cognitive control interactions and the role of human OFC in these functions, we studied patients with focal lesion to OFC using ERPs while they performed a computer-based test of executive functions, Executive Reaction Time (RT) Test, in the context of emotional distractors. This paradigm is designed to mimic everyday challenges in executive functions where simultaneous demands for multiple executive functions and unexpected emotional events meet. As patients with OFC lesion report challenges in executive functions in everyday situations but do not show deficits in traditional neuropsychological testing, we assumed that a paradigm introducing both emotional and cognitive challenge might be more sensitive than traditional tests in objectively capturing difficulties these patients encounter. The Executive RT Test has been shown to be sensitive in detecting emotional interference of task performance in healthy young subjects reflecting normal emotion-attention interaction (Hartikainen et al., 2012b; Erkkilä et al., submitted). Moreover, exaggerated attention capture by threat (i.e., altered emotion-attention interaction) has also been shown with this paradigm in patients with mild head injury and persistent symptoms (Hartikainen et al., 2010b) and in patients with refractory epilepsy treated with deep brain stimulation and vagus nerve stimulation (Hartikainen et al., 2014; Sun et al., 2015, 2017). In line with these previous studies, we focused on emotional modulation of late attentional and cognitive control phases reflected in N2 and P3 peaks and used N2P3 peak-to-peak amplitude as an electrophysiological biomarker for emotion-attention interaction. Furthermore, we assessed the subsequent emotional modulation of late positive potential (LPP), reflecting continued emotional processing after N2P3 potential.

For adaptive behaviors, efficient cognitive and attentional control is needed to either select the appropriate behavioral responses or suppress undue emotional reactions in face of emotional events. To that end, task-irrelevant emotional information compete for attentional and executive resources required to perform the task and thus task-irrelevant emotional events frequently interfere with performance in tasks requiring attention and executive functions in healthy subjects (Hartikainen et al., 2000, 2010a, 2012b; Hodsoll et al., 2011). In contrast to healthy subjects, we have previously shown that patients with lesion to the OFC show stronger than normal bias to voluntary attention supporting task performance but at the expense of involuntary attention allocation that might be beneficial outside the current task demands (Hartikainen et al., 2012a; Mäki-Marttunen et al., 2017). In a recent study with OFC lesion patients, non-emotional auditory stimuli evoked reduced amplitude of N1 potential which is known to be modulated by top-down attention control (Kam et al., 2018). Other electrophysiological studies have reported altered attentional processing of emotion and novelty after OFC lesion, although with equivocal results. Rule et al. (2002) found enhanced P3 potentials to aversive task-irrelevant somatosensory stimuli. Decreased P3 to novel irrelevant auditory stimuli along with normal P3b to targets has been previously reported in patients with OFC lesion (Løvstad et al., 2012b). In line with reduced attention-related ERPs to novel and emotional stimuli, task-irrelevant emotional photographs resulted in attenuated N2P3 peak-to-peak amplitudes in patients with OFC lesion whereas increased N2P3 amplitudes to immediately following targets were observed (Hartikainen and Knight, 2003; Hartikainen et al., 2012a). Enhanced N2P3 amplitudes were also observed in the context of task-relevant threat-related stimuli in OFC lesion (Mäki-Marttunen et al., 2017). These results suggest that the OFC has a role in guiding attention to emotionally or otherwise significant events even when irrelevant to the current task, modulating the extent of emotional impact on task-related attentional and cognitive control processes and in contributing to the balance between voluntary and involuntary attention especially in the context of emotion.

The areas involved in emotional processing, like the OFC and the amygdala, are thought to interact with the frontoparietal attention network, including the lateral prefrontal cortex, parietal cortex and the frontal eye fields (Pessoa, 2010), to allow for normal emotion-attention interaction. OFC evaluates the value and significance of emotional stimuli (Wright et al., 2008) and directs this information to other brain areas responsible for attentional control and executive functions. Attention modulates the value coding in OFC (Xie et al., 2018) and dopaminergic modulation of OFC has been shown to alter attentional performance (Winstanley et al., 2010). The posterior OFC has been shown to activate together with temporoparietal areas and the anterior cingulate cortex in response to salient events that occur outside the current focus of attention but require evaluation of potential behavioral relevance (Gruber et al., 2010). Thus, OFC may be part of the neural system that allows for monitoring the environment for potentially significant information even when outside the current task or focus of attention.

Intact communication between attentional and emotion-related networks, and their key nodes such as the OFC, is needed for appropriate emotion-attention interaction allowing for successful emotion-guided behaviors. Emotional and attentional interactions are multidirectional, intertwined and dynamic within sensory, limbic and attentional networks that interface in OFC. Thus, instead of assessing emotion-attention interaction as a static phenomenon in a single time point it is important to evaluate the temporal evolution and dynamics of this interaction in healthy subjects and how the dynamics are altered in patients with OFC lesion. ERPs with temporal resolution compatible with rapidly evolving mental events are suitable for such an approach. With this approach it is possible to gain information that eventually allows for better insight into deficits these patients encounter in real life situations that currently elude traditional assessment.

Normal emotional modulation of attention-related brain potentials is typically reflected in enhanced N2, P3 or N2-P3 potentials (Dennis and Chen, 2007; Olofsson et al., 2008; Mäki-Marttunen et al., 2015) or increased slow positivity during LPP (Hajcak et al., 2009) depending on the task. The observed impact of task-irrelevant emotional information on task-related attentional processes is typically lateralized to the right hemisphere dependent functions as well as target-related brain potentials over the right parietal region (Hartikainen et al., 2000, 2007, 2010a). In order to isolate the pure impact of emotion, the impact of visual stimuli can be subtracted by means of difference waveforms where an ERP evoked by a condition with emotionally neutral distractor is subtracted from an ERP evoked by a condition with threat-related emotional distractor with exactly the same basic physical features. Such difference waveforms reflect the mere impact of emotion with brain potentials related to visual processing subtracted (Hartikainen et al., 2007). In the current study, we used black line drawings of biologically relevant threat-related stimuli, i.e., spiders and emotionally neutral control images constructed from identical line components but in a different configuration that did not have emotional value. Such simple threat-related stimuli used in the current study are known to be prioritized for attention networks (Vuilleumier and Schwartz, 2001) and provide means to tap into potential alterations in emotion-attention interaction due to OFC lesion. Because the N2 potential reflects early cognitive control, particularly in a response inhibition/NoGo task (Donkers and van Boxtel, 2004; Megías et al., 2017), and the P3 potential reflects response inhibition and possibly response cancellation in a NoGo task (Kok et al., 2004; Randall and Smith, 2011; Groom and Cragg, 2015) as well as attention allocation (Polich, 2007), they are suitable candidates for studying the interaction of emotion and attention/cognitive control. We further combined these amplitudes to N2P3 peak-to-peak amplitude as our previous studies with clinical populations have suggested that in contrast to single peak measurements, N2P3 peak-to-peak amplitude may provide a more robust measure of attention (Mäki-Marttunen et al., 2015, 2017) and help control for abnormal EEG shifts and slow waves frequently observed in clinical populations.

In the current study, we aimed at assessing the impact of OFC lesion on the temporal dynamics of emotion-attention and emotion-cognitive control interaction. We assessed how task-irrelevant emotional distractors modulate the N2, P3 and LPP during a task requiring attention and cognitive control in healthy subjects and in patients with OFC lesion. In line with our previous studies (Hartikainen et al., 2007, 2010a), we expected healthy control participants to show right-lateralized modulation of attention-related ERPs to task-irrelevant emotion which would reflect normal emotion-attention interaction dominated by the right hemisphere. In comparison, we expected altered modulation of attention and cognitive control related ERP components in patients with OFC lesion. In addition, we studied whether patients with OFC lesion experience increased difficulties in everyday executive functions as previously reported (Løvstad et al., 2012a; Mäki-Marttunen et al., 2017) and assessed with the Behavior Rating Inventory of Executive Functions—Adult version (BRIEF-A; Roth et al., 2005) self-report questionnaire. We also assessed whether there is any objective evidence of executive dysfunction as reflected in performance in a computer-based Executive RT Test that engages several executive functions simultaneously in the context of threat-related distractors.

## Materials and Methods

### Subjects

Twelve patients (mean age = 58 years, male = 11, female = 1, mean years of education = 13) with acquired lesion to the OFC formed the OFC lesion group. Lesion etiologies were traumatic brain injury (*n* = 8), operated meningioma (*n* = 3) and aneurysmatic subarachnoidal hemorrhage (*n* = 1). All patients had participated in a previous study of our research group performing a modified version of the current executive function test. The Control group consisted of 12 neurologically healthy subjects (mean age = 53 years, male = 6, female = 6, mean years of education = 15) recruited as a convenience sample from subjects who had previously participated in a study of our research group, to reduce the effect of learning on between-group differences. The groups did not differ significantly in terms of age and years of education. However, subject sex distribution was not balanced as there was only one female in the OFC lesion group and six females in the Control group. General exclusion criteria for both groups included history of substance abuse, previous neurological disorder (such as ADHD), and current moderate or severe depression. The study was approved by the Ethical Committee of the Tampere University Hospital and participants provided their written informed consent according to the Declaration of Helsinki governing the use of human subjects.

**Figure 1:**
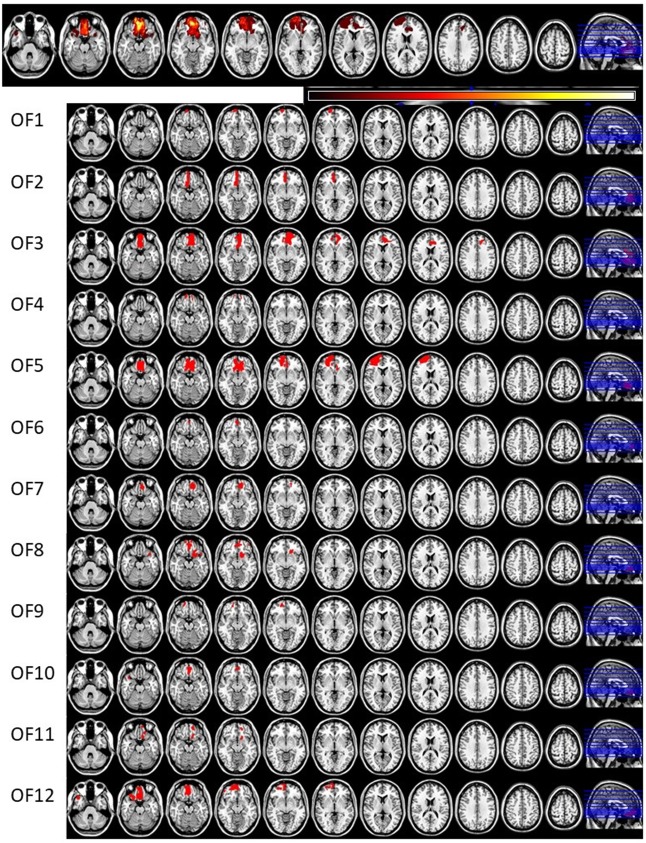
Lesion reconstructions of the orbitofrontal cortex lesion group. Eleven horizontal slices are presented for each patient. The top panel represents group overlay of all lesions, where the color bar indicates the number of patients having the lesion on the same area, with darker colors indicating fewer patients and lighter colors representing more patients. In the MRI lesion reconstruction images of single patients the red color indicates the lesion location.

Invitations to participate in the study were based on neuroradiological evaluations of suitable lesion location by an experienced neuroradiologist who referred patients to the research group. Lesion characterization was based on magnetic resonance imaging (MRI) except for one patient whose MRI scan was unavailable. This patient’s lesion evaluation was determined by computed tomography (CT) scan. Lesion location and size were subsequently evaluated by a neurologist, and patients with multiple or extensive lesions extending beyond the OFC were excluded from the study. The most serious injury class in this study was moderate brain injury, based on the Finnish diagnostic guidelines for brain injuries (Brain Injuries. Current Care Guidelines, 2017). Moderate brain injury in Finnish classification corresponds to mild complicated or moderate brain injury in brain injury literature (Williams et al., 1990) and in American diagnostics guidelines (Department of Veterans Affairs and Department of Defense, 2016). Lesion reconstructions were carried out using MRIcron version 11 (Rorden et al., 2007) and are presented in Figure 1. Lesion characteristics, including type of injury, size and location, are presented in Table 1.

**Table 1:** Lesion characteristics of the orbitofrontal cortex lesion group.

Subject	Etiology of injury	Time since injury (months)	Lesion size (cm^3^)	Lesion side	Brodmann areas
OF1	Traumatic brain injury	36	2.94	Right	10, 11
OF2	Subarachnoidal hemorrhage	120	11.58	Right	10, 11, 25, 32
OF3	Operated meningioma	40	34.25	Both	9, 10, 11, 24, 25, 32, 45, 46, 47, 48
OF4	Traumatic brain injury	39	2.43	Both	11, 20, 36, 38
OF5	Operated meningioma	41	49.29	Both	9, 10, 11, 25, 32, 45, 46, 47, 48
OF6	Traumatic brain injury	183	1.36	Right	10, 11
OF7	Operated meningioma	71	4.34	Left	10, 11
OF8	Traumatic brain injury	24	10.65	Both	10, 11, 20, 25, 28, 34, 38, 46, 47, 48
OF9	Traumatic brain injury	46	1.57	Right	10, 11
OF10	Traumatic brain injury	24	3.62	Both	10, 11, 20, 38
OF11	Traumatic brain injury	19	2.99	Left	11, 25, 48
OF12	Traumatic brain injury	46	19.13	Both	10, 11, 20, 21, 25, 34, 36, 38, 46, 47, 48
**Mean**		**57.4**	**12.0**		

*Lesion etiology, side, size in cubic centimeters, affected Brodmann areas and time from the injury in months are presented*.

### Questionnaires

BRIEF-A (Roth et al., 2005) was used to assess participants’ subjective judgment of their executive functions in daily life. The questionnaire presents 70 statements concerning different situations employing executive functions requiring the responder to assess whether he/she exhibits the kind of behavior “never,” “sometimes” or “often.” Nine different aspects of executive functions are assessed (Inhibition, Shifting, Emotional Control, Self-Monitoring, Initiation, Working Memory, Planning/Organizing, Task Monitoring, Organization of Materials) and later combined to produce three summary indices (Behavioral Regulation Index (BRI), Metacognition Index (MI) and Global Executive Composite (GEC)). The BRIEF-A is suitable for assessing self-reported executive dysfunction in brain injured patients (Waid-Ebbs et al., 2012). Beck’s Depression Inventory (BDI; Beck et al., 1996) was used to measure possible depressive symptoms of participants because depression was one of the exclusion criteria and could impair task performance (Austin et al., 2001) and bias attention allocation to negative emotional stimuli (Gotlib et al., 2004). Rivermead Post-Concussion Symptoms Questionnaire (RPQ; King et al., 1995) was used to measure the amount of post-concussion related symptoms in patients and determine whether they were still symptomatic. Participants also completed a basic demographic information questionnaire.

### Executive Reaction Time Test

**Figure 2:**
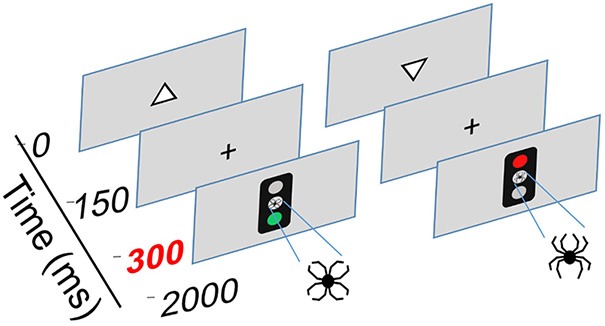
Schematic illustration of the Executive Reaction Time Test by Hartikainen et al. (2010b), an integrated test of executive functions with task-irrelevant emotional distractors. This test mimics everyday demands for executive functions as it requires multiple different executive functions to be engaged simultaneously, including working memory, response inhibition and the ability to change behavioral sets flexibly. Corresponding to real-life situations where successful behavior requires sharing cognitive control resources between the current task and intervening emotional events, the test requires cognitive control to sufficiently control emotional interference in order to perform well. Thus, this test allows for sensitive assessment of executive functions as well as emotion-attention and emotion-executive function interaction. Each trial begins with a white triangle appearing on the screen pointing either upwards or downwards. The participants must attend to the pointing direction of the triangle and keep it in working memory. A Go or a NoGo signal in form of a traffic light is presented 150 ms after the offset of the triangle in the middle of the screen. The color of the traffic light signals whether the participant is supposed to respond or withhold from responding; green light = Go and red light = NoGo. In half of the blocks the traffic light rule for responding is reversed requiring the subject to flexibly change sets and respond according to a new rule. In Go-condition, participants were instructed to press a response pad button corresponding to the triangle orientation memorized (triangle up = middle finger, triangle down = index finger). Task-irrelevant emotional distractors were presented in the middle position of the traffic light. The emotional distractors were composed of identical line-elements but in a different configuration forming either a figure of a spider (negative, threatening distractor) or a flower (neutral distractor).

The Executive RT Test, developed by Hartikainen et al. (2010b) is a computer-based Go/NoGo task that incorporates non-emotional target stimuli and emotion-related (neutral and threatening) irrelevant distractor stimuli. The task requires several types of executive functions, including response inhibition, set shifting and updating, working memory and selective attention. Schematic diagram and task description of the Executive RT Test are presented in Figure 2.

Participants performed the Executive RT Test while seated approximately one meter away from a computer screen in a sound attenuated booth. They were instructed to react as fast and as accurately as possible to the orientation of the triangle. The emotional figure served as an irrelevant distractor, thus the participants need not consciously react to it, but it may capture attentional resources via bottom-up mechanism and thereby create attentional competition. Three error types are possible in performing the test: Miss, i.e., missing the button press although button press was required; Incorrect button press, i.e., pressing the wrong button, for example pressing down button even though the triangle was pointing upwards; and Commission error, i.e., pressing the button even though one was required to withdraw from responding (the so called “NoGo-error”). Misses reflect problems in initiating a response or lapses in attention, Incorrect button presses reflect lapses in working memory, while Commission errors indicate problems in response inhibition. In summary, the task requires efficient control of executive functions, selective attention and tests the effect of task-irrelevant emotional stimuli on these processes and the ability of the subjects to control for it.

The number of Go/NoGo blocks, threat-related and neutral distractors and orientation of the triangle were all balanced to a 50:50 ratio and presented in random order. Each block consisted of 64 trials and the total length of the task was 16 blocks, resulting in 1,024 trials per participant. Half of the blocks were performed using the right hand and the other half performed using the left hand. RT and number of errors served as measures of task performance. The emotional figures were composed of identical black lines in order to control for physical properties (size, color, complexity, luminance) and prevent stimuli properties other than emotional content from influencing visual attention and ERPs.

### EEG Recording and Preprocessing

The EEG signal was recorded using 64 Ag/AgCl active electrodes (actiCAP, Gilching, Germany) along with a QuickAmp-amplifier system and Brain Vision Recorder software (Brain Products, GmbH). The sampling rate used to digitize EEG was 500 Hz. Electrode impedances were kept below 5 kΩ throughout the recording. The EEG preprocessing and construction of ERPs was done offline using Brain Vision Analyzer 2 software (Brain Products, GmbH). The EEG was down sampled to 250 Hz and filtered with IIR filters to 0.01–70 Hz followed by blink artifact removal by semiautomatic, independent component analysis–based function, method described by Jung et al. (2000). An additional artifact removal was performed removing intervals with more than 100 μV voltage difference to the surrounding signal. The data was then re-referenced to the linked right and left lobules auriculae and further filtered to 0.01–30 Hz before segmentation. Segmentation to create ERPs was performed by cutting segments starting 200 ms before trial onset, i.e., the appearance of the triangle on the screen, and ending 1,800 ms after. Segments were baseline-corrected to the base line of a timeframe from before 200 ms to the trial onset.

The ERP segments were averaged based on condition (Go or NoGo) and distractor type (Emotional, Neutral), resulting in four different ERP conditions for each subject (Go Emotional, Go Neutral, NoGo Emotional and NoGo Neutral, respectively). The minimum cut-off for the number of segments per condition per participant was 50. Each trial began with the triangle, i.e., located at timepoint 0 ms. Go/NoGo signal, i.e., the traffic light, appeared 300 ms after the trial onset. The N2 and P3 components appearing after the Go/NoGo signal were identified from the Grand Average waveforms based on visual inspection and semiautomatic peak detection based on the timeframes defined by visual inspection. The N2 was defined as the most negative peak in a time frame ranging from 450 ms to 670 ms (i.e., 150–370 ms from the traffic light cue) and the P3 as the most positive peak in a time frame from 600 ms to 900 ms (i.e., 300–600 ms from the traffic light cue). We exported the mean value around the observed peak ±5 time points from the peak marker for analysis. N2 amplitude is normally well depicted in frontocentral regions whereas the target-evoked P3 amplitude is seen on the parietal areas and we included electrodes that best capture these components. For this reason and in order to reduce the number of statistical comparisons and to keep methodology similar to our previous studies (Mäki-Marttunen et al., 2015, 2017), we selected one frontal, central and parietal electrode over each hemisphere for statistical analysis: F3 (left frontal), F4 (right frontal), C3 (left central), C4 (right central), P3 (left parietal) and P4 (right parietal). The N2P3 peak-to-peak amplitude was constructed by subtracting the N2 amplitude from the P3 amplitude for each electrode.

### Statistical Analysis

#### Behavioral Analysis

The behavioral analysis was performed using R version 3.3.3 (R Core Team, 2017). The distribution of RTs was skewed and they were normalized using logarithmic transformation before the analysis. RT analysis was conducted with mixed model ANOVA where Group (OFC, Control) served as a between-group factor and Emotion (Emotional, Neutral) as a within-subjects factor. Error analysis was done using generalized binary logistic regression as suggested by Jaeger (2008) and Dixon (2008). In the binary logistic regression model, Group (OFC, Control) and Emotion (Emotional, Neutral) were used as fixed effect predictors and Subject as a random effect predictor. Subject was classified as a member of the OFC group or the Control group in a hierarchical manner.

For the binary logistic regression analysis, error data was dichotomized. Three types of errors were possible (See “Executive Reaction Time Test” section); Go-errors, i.e., “Incorrect button press” and “Miss,” and NoGo-errors, i.e., “Commission errors.” Incorrect button presses were dichotomized as either “incorrect” or “other” (i.e “correct” or “miss,” other possible answers in a Go-situation) and Misses as “miss” or “other” (i.e., “correct” or “incorrect” using a similar logic as previously). Commission errors were dichotomized as “commission error” or “correct” (no other error types available in NoGo-situation). Total errors were labeled as “error” or “correct.” Following this, a separate model to predict probability to make an error was created for each condition. We used the “lme4” package version 1.1–13 (Bates et al., 2015) for binary logistic regression modeling and analysis.

Before modeling, the data was checked for outliers. A subject was considered an outlier if his/her error sum in any error category exceeded the group mean error rate for that error category by more than 2.5 standard deviations (SD). In case of an outlier, the data was analyzed without the outlier and if the results changed, the outlier was excluded from the final analysis. Data was also checked for outlier and “wrong-rule” blocks. A block with wrong rule was a block where the subject apparently answered using the wrong answering rule. If 75% of the answers were Commission errors and Misses, the block was considered a wrong rule block and excluded. Outlier block was a block where the subject’s error rate was more than three SD above his/her mean error rate. Neither wrong rule blocks nor outlier blocks were detected in the data, i.e., no blocks were excluded from the analysis. Outlier subject criteria were met several times in both groups, however, the result was affected only once. Participant number 12 from the Control group was excluded from further behavioral and neurophysiological analysis based on the higher amount of total errors compared to the rest of the group (Total errors, group mean = 3% vs. participant 12 mean = 9.6%).

#### ERP and ERP Difference Wave Analysis

The N2P3 peak-to-peak amplitude was used for statistical analysis. We used mixed model ANOVA (repeated measures and between-group measures) to compare between-group and within-subjects factors simultaneously. Group (OFC, Control) was defined as a between-group factor and Emotion (Emotional, Neutral), Laterality (Right, Left) and Region (Frontal, Central, Parietal) as within-subjects factors. ERPs were analyzed separately for the Go- and NoGo-conditions. Data suitability to ANOVA assumptions was tested, normality tests yielding normal or close to normal distributions.

To analyze differences in attention allocation to emotion between the groups and to eliminate the effect of other visual processes and potential artifacts on the observed differences, we created difference waveforms by subtracting ERP amplitude in context of neutral distractor from ERP amplitude in context of emotional distractor in both groups (ERP Emotional—ERP Neutral). This difference waveform was used to investigate differences in continuous emotional processing in selected time windows and subjected to separate statistical analysis. In Go-situation we chose time windows corresponding to the time of P3 peak and the following slow positive waveform, the LPP, which is reported to be larger when emotional stimuli is only attended to but reduced with successful reappraisal of emotion (Hajcak and Nieuwenhuis, 2006), i.e., 700–800 ms and 800–900 ms in our paradigm. In NoGo-situation we chose time windows around the N2 peak and the P3 peak, i.e., 600–700 ms and 700–800 ms, for further analysis as these potentials are thought to reflect different phases of cognitive control required for response inhibition and are known to be modulated by emotion with the extent of modulation reflecting factors influencing emotion-cognition interaction such as emotional intelligence (Megías et al., 2017). The amount of selected time windows was kept to minimum to control for familywise error rate. Subtraction ERP emotional—ERP Neutral was conducted first and the mean amplitude in the aforementioned 100 ms time windows exported for statistical analysis. The difference waveforms reflecting mere impact of emotion were subjected to ANOVA where factor Group (OFC, Control) served as a between-group factor and Laterality (Right, Left) and Region (Frontal, Central, Parietal) as within-subjects factors.

*Post hoc* analysis with ANOVA was performed when significant interactions were met. When decomposing interactions for *post hoc* ANOVAs, we chose to adjust the significance level based on the Bonferroni method, to *p* = 0.017 on the final ANOVA level to correct for multiple comparisons. Statistical analysis was performed using R version 3.3.3 (R Core Team, 2017) and the package “ez” version 4.4-0 for ANOVA comparisons (Lawrence, 2016). Sphericity corrections were applied whenever non-spherical data was encountered.

#### Questionnaire Analysis

The BRIEF-A composite scores and indices, the RPQ subscores and total scores, and the BDI scores were analyzed using R version 3.3.3 (R Core Team, 2017). Normality tests resulted in a non-normal distribution in most cases, thus Wilcoxon rank-sum tests for nonparametric comparisons were applied using package coin (Hothorn et al., 2006, 2008). BRIEF-A raw scores were transformed to normative *t*-scores and the *t*-scores used for between-groups comparison, as they allow comparison of a standard coeval sample (Roth et al., 2005). From the RPQ we compared the current reported symptoms between the groups. The validity of the RPQ total score has been questioned because post-concussion symptoms are nonspecific, fitting many other conditions as well. Dividing the total score to emotional, somatic and cognitive symptom categories has been suggested in several studies, thus we divided the total score into those categories according to Smith-Seemiller et al. (2003) and Potter et al. (2006), and analyzed them separately.

## Results

### Task Performance

RT analysis resulted in no significant main effects or interactions; there was no difference between the group RTs (OFC lesion group, RT = 476.87 ms ± 191.61 ms vs. Control group RT = 459.09 ms ± 180.16 ms). The OFC lesion group was 2.3 times more likely to commit an error of any type compared to the Controls (Total errors, Main effect of Group, OFC vs. Controls: OR 0.43, (95% CI = 0.21–0.88), 4.7% vs. 2.1%). The OFC lesion group was 5.2 times more likely to miss a response compared to the Control group (Miss, Main effect of Group, OFC vs. Controls: OR 0.19, (95% CI = 0.041–0.90), 0.9% vs. 0.2%). The increased probability to commit an error of any type or miss responding in the OFC lesion group was not dependent on the emotional distractor. The probability to commit an Incorrect button press was almost significantly different (*p* = 0.059) between the groups, the OFC lesion group committing more Incorrect button presses (Main effect of Group, OFC vs. Controls: OR 0.39, (95% CI = 0.15–1.04), 2.9% vs. 1.2%). Binary logistic regression for Commission errors did not yield significant results.

### ERPs

#### Go-Condition

The mean amplitudes and SD for the N2 and P3 ERP components as well as the N2P3 peak-to-peak amplitudes for both groups in each condition are listed in Table 2. In the Go-condition, analysis of the N2P3 peak-to-peak amplitude yielded no statistically significant main effects. There was a significant interaction of Group × Emotion (*F*_(1,21)_ = 10.36, *p* = 0.0041, $\eta_{\text{G}}^{2}$ = 0.0026) which was investigated further by dividing the data by Group and analyzing them separately with *post hoc* ANOVAs. In the Control group, the N2P3 amplitude in context of the emotional distractor was larger compared to the neutral distractor (*F*_(1,10)_ = 6.31, *p* = 0.031, $\eta_{\text{G}}^{2}$ = 0.0031; Emotional = 7.10 μV ± 3.74 μV vs. Neutral = 6.71 μV ± 3.59 μV). However, under the Bonferroni-adjusted significance criteria this effect was only approaching significance. In the OFC lesion group, the valence of the distractor had no significant effect on the N2P3 amplitude (*F*_(1,11)_ = 4.49, *p* = 0.058, $\eta_{\text{G}}^{2}$ = 0.0031, Emotional = 8.25 μV ± 3.39 μV vs. Neutral = 8.64 μV ± 3.74 μV).

**Table 2:** N2, P3 and N2P3 amplitudes and standard deviations (in μV) presented for each condition (Go and NoGo), for both groups and separately for both emotional distractors and for each electrode over the left and right frontal (F3, F4), central (C3, C4) and parietal (P3, P4) scalp sites used in the analysis.

Frontal			F3 (left)			F4 (right)		
Condition	Group	Distractor	N2	P3	N2P3	N2	P3	N2P3
Go	Control	Emotional	−5.3 (3.8)	1.5 (3.2)	6.8 (3.3)	−3.9 (4.4)	3.1 (2.8)	7.0 (3.3)
		Neutral	−5.1 (3.8)	1.4 (3.1)	6.5 (3.3)	−3.6 (4.7)	2.8 (3.0)	6.4 (3.0)
	OFC	Emotional	−3.5 (6.0)	4.4 (7.2)	7.9 (4.0)	−2.5 (4.9)	5.6 (5.2)	8.2 (3.5)
		Neutral	−3.6 (5.6)	4.7 (7.0)	8.3 (4.7)	−2.8 (4.8)	5.7 (5.3)	8.4 (4.4)
NoGo	Control	Emotional	−3.0 (2.1)	7.4 (4.2)	10.4 (4.5)	−3.0 (2.3)	6.3 (3.8)	9.3 (4.6)
		Neutral	−2.6 (2.7)	7.1 (3.9)	9.7 (4.1)	−2.8 (2.9)	5.8 (3.7)	8.5 (4.1)
	OFC	Emotional	−2.3 (3.6)	10.4 (4.8)	12.7 (5.5)	−2.6 (3.9)	10.3 (4.9)	12.8 (5.2)
		Neutral	−1.9 (3.7)	10.1 (4.4)	12.0 (5.2)	−1.9 (3.3)	10.2 (4.7)	12.0 (5.0)
**Central**			**C3 (left)**			**C4 (right)**		
**Condition**	**Group**	**Distractor**	**N2**	**P3**	**N2P3**	**N2**	**P3**	**N2P3**
Go	Control	Emotional	−6.5 (3.9)	0.7 (3.0)	7.2 (4.0)	−4.3 (3.2)	3.2 (4.3)	7.5 (4.3)
		Neutral	−6.5 (3.9)	0.6 (3.1)	7.1 (3.8)	−4.3 (3.5)	2.7 (3.8)	7.1 (4.0)
	OFC	Emotional	−4.7 (6.4)	3.4 (6.3)	8.2 (3.8)	−4.1 (6.1)	4.5 (4.8)	8.6 (4.4)
		Neutral	−4.8 (6.2)	3.5 (6.1)	8.4 (4.2)	−4.6 (5.8)	4.9 (5.0)	9.5 (4.6)
NoGo	Control	Emotional	−2.1 (2.2)	7.6 (3.9)	9.6 (4.0)	−1.8 (1.7)	7.0 (4.1)	8.7 (4.6)
		Neutral	−1.9 (2.3)	7.1 (3.8)	9.0 (3.9)	−1.6 (1.7)	6.5 (4.0)	8.1 (4.3)
	OFC	Emotional	−1.5 (3.8)	9.1 (3.6)	10.7 (5.0)	−2.0 (3.7)	9.1 (4.5)	11.1 (4.8)
		Neutral	−1.1 (4.3)	9.2 (3.6)	10.3 (5.1)	−1.2 (3.8)	8.9 (4.4)	10.1 (5.0)
**Parietal**			**P3 (left)**			**P4 (right)**		
**Condition**	**Group**	**Distractor**	**N2**	**P3**	**N2P3**	**N2**	**P3**	**N2P3**
Go	Control	Emotional	−2.8 (4.7)	4.4 (3.6)	7.2 (4.7)	−1.4 (4.3)	5.5 (3.8)	6.9 (4.6)
		Neutral	−2.9 (5.1)	4.0 (4.0)	6.9 (4.8)	−1.2 (4.8)	5.0 (4.1)	6.2 (4.5)
	OFC	Emotional	−2.7 (4.4)	5.6 (4.4)	8.3 (3.2)	−2.6 (3.7)	5.9 (3.4)	8.5 (3.8)
		Neutral	−3.2 (4.5)	5.2 (4.0)	8.4 (3.1)	−2.8 (3.8)	6.0 (3.3)	8.8 (3.7)
NoGo	Control	Emotional	−0.7 (3.7)	6.1 (4.6)	6.9 (4.0)	−0.4 (3.8)	6.4 (4.0)	6.8 (4.6)
		Neutral	−0.9 (4.0)	6.2 (3.9)	7.1 (3.8)	−0.5 (3.8)	6.4 (3.8)	6.9 (4.3)
	OFC	Emotional	−0.9 (3.4)	7.2 (3.5)	8.1 (3.8)	−0.4 (2.5)	7.5 (3.6)	7.9 (3.1)
		Neutral	−0.7 (4.1)	7.0 (3.6)	7.7 (4.2)	−0.3 (2.8)	7.4 (3.4)	7.6 (3.2)

**Figure 3:**
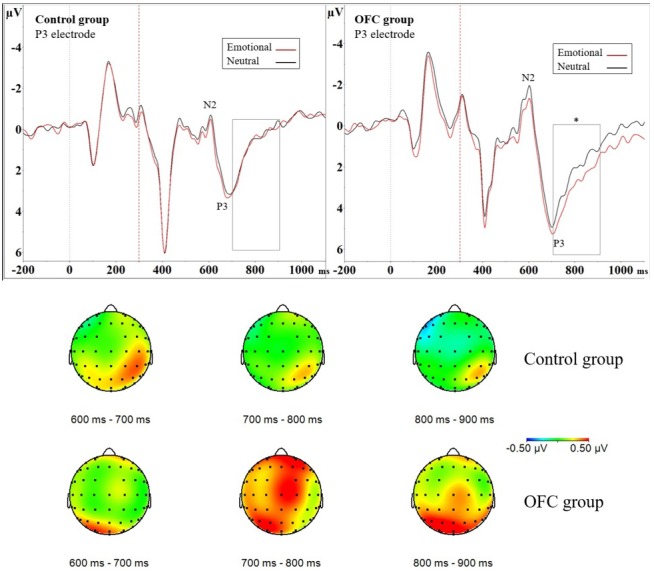
Greater modulation of late positive potential (LPP) by threat due to orbitofrontal lesion. Above event-related potential (ERP) waveforms illustrate N2P3 complex and the following LPP in the Control group (ERP on the left) and in the OFC lesion group (ERP on the right) in the P3 electrode. Significantly enhanced and prolonged positivity was detected due to threat-related emotional distractors in the OFC lesion group but not in the Control group in the 700–900 ms time window depicted with a rectangle. Statistical significance is marked with an asterisk. Dashed red line at 300 ms represents onset of the response cue (i.e., the traffic light). Below topography of the difference waveform isolating emotional modulation of brain activity (ERP Emotional—ERP Neutral) for three subsequent 100 ms time windows in each group. Time range 700–900 ms shows increased left-lateralized positivity on parietal region in the OFC lesion group (lower row) in contrast to Control group (upper row). In the Control group the increased positivity to emotion detected in analysis of the N2P3 peak-to-peak amplitude was more focal, right-lateralized and limited in time (topography time window 600–700 ms) as opposed to the OFC lesion group, who exhibited more diffuse, left-lateralized and prolonged positivity.

There was also an interaction effect of Group × Emotion × Laterality (*F*_(1,21)_ = 4.48, *p* = 0.046, $\eta_{\text{G}}^{2}$ = 0.0004). The data was divided by Group and the groups analyzed separately. *Post hoc* ANOVA revealed significant interaction effect of Emotion × Laterality in the Control group (*F*_(1,10)_ = 9.19, *p* = 0.013, $\eta_{\text{G}}^{2}$ = 0.0006) but not in the OFC lesion group (*F*_(1,11)_ = 1.04, *p* = 0.33, $\eta_{\text{G}}^{2}$ = 0.0003). Further analysis of the interaction was performed by dividing Control group data by Laterality and conducting separate *post hoc* ANOVAs on the right and left hemispheres, revealing N2P3 amplitude was larger in context of the emotional distractor over the right hemisphere (*F*_(1,10)_ = 8.47, *p* = 0.016, $\eta_{\text{G}}^{2}$ = 0.0061; Emotional = 7.13 μV ± 3.89 μV vs. Neutral = 6.57 μV ± 3.64 μV; Figure 3). The valence of the distractor had no significant effect on the N2P3 amplitude over the left hemisphere in the Control group (*F*_(1,10)_ = 2.78, *p* = 0.13, $\eta_{\text{G}}^{2}$ = 0.0010). There was also an interaction effect of Group × Emotion × Laterality × Region (*F*_(2,42)_ = 3.45, *p* = 0.041, $\eta_{\text{G}}^{2}$ = 0.0001). In addition to the aforementioned effects in the Control group, decomposing this interaction by *post hoc* ANOVAs revealed an interaction of Emotion × Region × Laterality in the OFC lesion group (*F*_(2,22)_ = 4.14, *p* = 0.03, $\eta_{\text{G}}^{2}$ = 0.0005). The OFC lesion group data was divided by Laterality and *post hoc* ANOVAs performed, however, no further significance was detected on either hemisphere.

#### NoGo-Condition

In the NoGo-condition, analysis of the N2P3 peak-to-peak amplitude showed Main effect of Emotion (*F*_(1,21)_ = 8.64, *p* = 0.008, $\eta_{\text{G}}^{2}$ = 0.0033) and Main effect of Region (*F*_(2,42)_ = 37.4, *p* < 0.001, $\eta_{\text{G}}^{2}$ = 0.11) but no statistically significant difference between the groups. Main effect of Emotion indicated that the N2P3 amplitude in context of the emotional distractor was larger than N2P3 amplitude in context of the neutral distractor (Emotional = 9.62 μV ± 4.76 μV vs. Neutral = 9.13 μV ± 4.55 μV). Main effect of region indicated that the size of the N2P3 amplitude differed significantly between each brain region, being largest on the frontal region and smallest on the parietal region (*post hoc* *t*-test Frontal vs. Central, *p* < 0.001, Frontal vs. Parietal, *p* < 0.001 and Central vs. Parietal, *p* < 0.001; Frontal = 11.0 μV ± 4.83 μV vs. Central = 9.74 μV ± 4.47 μV vs. Parietal = 7.39 μV ± 3.70 μV). A significant interaction of Emotion × Region was also observed (*F*_(2,42)_ = 5.31, *p* = 0.009, $\eta_{\text{G}}^{2}$ = 0.0011). *Post hoc* ANOVAs were performed for each region separately, revealing larger N2P3 amplitudes in context of the emotional distractor on frontal and central regions but not on the parietal region (Frontal region: *F*_(1,22)_ = 14.78, *p* = 0.0009, $\eta_{\text{G}}^{2}$ = 0.0060; Emotional = 11.36 μV ± 4.99 μV vs. Neutral = 10.63 μV ± 4.71 μV. Central region: *F*_(1,22)_ = 13.27, *p* = 0.0014, $\eta_{\text{G}}^{2}$ = 0.0054; Emotional = 10.06 μV ± 4.49 μV vs. Neutral = 9.42 μV ± 4.49 μV), supporting the observed main effects.

### ERP Difference Waveform Window Analysis

#### Go-Condition

In addition to assessing the impact of emotional distractors on attention-related ERP peaks with peak-to-peak analysis, we assessed the impact of emotion on selected 100 ms time windows. We isolated the impact of mere emotional value with ERP difference waveform (ERP Emotional Go—ERP Neutral Go) and analyzed the mean amplitude of the difference waveform within a 700–800 ms time window. This analysis resulted in no main effects, but an interaction effect of Group × Laterality × Region (*F*_(2,42)_ = 3.51, *p* = 0.039, $\eta_{\text{G}}^{2}$ = 0.024) was observed. To investigate this interaction further, the data was divided by Group and groups were analyzed separately with *post hoc* ANOVAs. In the OFC lesion group, *post hoc* ANOVA revealed Laterality × Region interaction (*F*_(2,22)_ = 4.75, *p* = 0.019, $\eta_{\text{G}}^{2}$ = 0.055). *Post hoc* ANOVAs conducted separately for each region showed that over the parietal region, the mean amplitude of the difference waveform significantly differed between the two hemispheres (*F*_(1,11)_ = 13.15, *p* = 0.004, $\eta_{\text{G}}^{2}$ = 0.16, Left Parietal = 0.61 μV ± 0.83 μV vs. Right Parietal = −0.019 μV ± 0.67 μV). In the OFC group emotional stimuli were associated with greater positivity over the left parietal cortex and the amplitudes in context of emotional compared to neutral distractors differed on the left parietal region (Figure 3). In the Control group, there were no main effects or interactions within this time window.

ERP difference waveform analysis of the mean amplitude within time window 800–900 ms resulted in Main effect of Region (*F*_(2,42)_ = 3.58, *p* = 0.037, $\eta_{\text{G}}^{2}$ = 0.036), indicating emotional modulation of brain activity differed significantly between the central and parietal regions (*post hoc* *t*-test Central vs. Parietal, *p* = 0.031; Parietal = 0.33 μV ± 0.68 μV vs. Central = −0.029 μV ± 0.75 μV). Furthermore, interaction effect Group × Laterality × Region (*F*_(2,42)_ = 5.38, *p* = 0.008, $\eta_{\text{G}}^{2}$ = 0.019) was observed and the data was divided by Group and groups analyzed were separately with *post hoc* ANOVAs. In the OFC lesion group, Main effect of Region (*F*_(2,22)_ = 5.42, *p* = 0.012, $\eta_{\text{G}}^{2}$ = 0.064) showed that the mean amplitude of the difference waveform within the analyzed time window differed significantly on the frontal and parietal regions (*post hoc* *t*-test Frontal vs. Parietal, *p* = 0.043; Frontal = −0.08 μV ± 0.84 μV vs. Parietal = 0.41 μV ± 0.84 μV) but not on the other regions (Frontal vs. Central, *p* = 0.93; Central vs, Parietal, *p* = 0.07). Interaction effect Laterality × Region was also observed (*F*_(2,22)_ = 5.96, *p* = 0.009, $\eta_{\text{G}}^{2}$ = 0.030). *Post hoc* ANOVAs performed separately for each region showed that the mean amplitude on the left parietal region was significantly larger compared to the mean amplitude on the right parietal region (*F*_(1,11)_ = 8.71, *p* = 0.013, $\eta_{\text{G}}^{2}$ = 0.072; Left Parietal = 0.65 μV ± 0.89 μV vs. Right Parietal = 0.18 μV ± 0.89 μV). There was also a trend, under the Bonferroni-adjusted significance criteria, towards a difference in the mean amplitudes on the right frontal and left frontal region (*F*_(1,11)_ = 5.16, *p* = 0.044, $\eta_{\text{G}}^{2}$ = 0.024; Right Frontal = 0.05 μV ± 0.80 μV vs. Left Frontal = −0.21 μV ± 0.92 μV). Inspection of the original ERP waveforms showed prolonged and enhanced LPP over the left parietal region in OFC group in context of emotional distractors (Figure 3). In the Control group, no further Main effects or Interactions were detected.

#### NoGo-Condition

**Figure 4:**
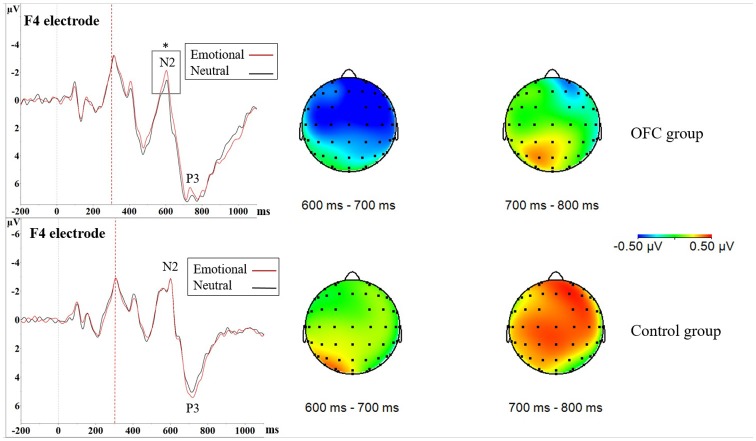
Increased NoGo N2 amplitude due to threat in orbitofrontal lesion. In Nogo-situation, both groups showed increased N2P3 amplitudes towards the emotional distractor, especially on the frontal and central cortices. The increase in the N2P3 amplitude in context of emotional distractor (red line) was located around the N2 peak in the OFC lesion group (upper figure) and around the P3 peak in the Control group (lower figure) as seen in the ERPs. Statistical significance is marked with an asterisk. Dashed red line at 300 ms represents onset of the stimuli (i.e., the traffic light). Time window analysis of the amplitude differences and separate N2 peak analysis further supported these findings. As depicted in the amplitude difference topographies there was greater negativity in the OFC lesion group (upper row) in time window 600–700 ms, corresponding to the N2 peak, whereas the Control group (lower row) exhibited greater positivity in time window 700–800 ms corresponding to the P3 peak.

In NoGo-situation, analysis of the mean amplitude of the difference waveform reflecting emotional modulation of brain activity during a response inhibition task (ERP Emotional NoGo—ERP Neutral NoGo) in 600–700 ms time window resulted in Main effect of Group (*F*_(1,21)_ = 4.53, *p* = 0.045, $\eta_{\text{G}}^{2}$ = 0.11; OFC = −0.50 μV ± 0.66 μV vs. Control = 0.18 μV ± 0.88 μV), with topography showing pronounced negativity in the OFC lesion group (Figure 4). As this time window is located close to the N2 peak, more negative values stand for larger amplitudes. Visual inspection of the grand average ERP waveforms in the OFC group showed the N2 amplitude in context of emotion was larger compared to the N2 amplitude in context of neutral stimuli. Due to these findings and theoretical interest on OFC’s role in contributing to emotional modulation of cognitive control, we decided to conduct an additional analysis of the N2 peak amplitude in both groups separately where Emotion, Region and Laterality served as within-subjects factors. In the OFC lesion group, there was Main effect of Emotion (*F*_(1,11)_ = 5.46, *p* = 0.039, $\eta_{\text{G}}^{2}$ = 0.0040), indicating that the N2 peak in context of the emotional distractor was larger (i.e., more negative) compared to the N2 peak in context of the neutral distractor (Emotional = −1.62 μV ± 3.03 μV vs. Neutral = −1.17 μV ± 3.27 μV). In the Control group, no such effects were detected (Main effect of Emotion, *F*_(1,10)_ = 0.17, *p* = 0.69, $\eta_{\text{G}}^{2}$ = 0.0004; Figure 4).

Difference waveform in 700–800 ms time window also yielded Main effect of Group (*F*_(1,21)_ = 5.02, *p* = 0.036, $\eta_{\text{G}}^{2}$ = 0.11; Control = 0.50 μV ± 0.72 μV vs. OFC = −0.20 μV ± 0.76 μV), topography showing pronounced positivity in the Control group (Figure 4). As this time window mostly corresponds to area around the P3 peak, more positive values indicate larger amplitudes. Visual inspection of the grand average ERP waveforms showed larger P3 peaks in context of emotion compared to neutral stimuli in the Control group. Due to these findings and theoretical interest in different roles of the N2 and the P3 peak in response inhibition, we conducted an additional analysis of the P3 peak in both groups separately, however, it yielded no significance related to emotion (Figure 4).

### Questionnaires

In the BRIEF-A the groups differed significantly in the amount of total executive function problems reported, with the OFC lesion group reporting more problems in the General Executive Composite score (GEC; OFC = 61.27 ± 13.64 points vs. Controls = 49.17 ± 9.68 points, *p* = 0.026). In the index scores the OFC lesion group reported more problems in Inhibit, Shift, Initiate, Working Memory and Plan/Organize. Scores, SD and *p*-values are presented in Table 3. From the two summary indices, the groups did not differ in the Behavior Regulatory Index but the OFC lesion group scored higher in the MI (OFC = 62.0 ± 12.08 points vs. Controls = 48.58 ± 9.23 points, *p* = 0.007). There was no statistically significant difference between the groups in Emotional Control. BDI showed no statistically significant differences between the groups in depressive symptoms. In the RPQ, the groups differed significantly in how much they reported overall post-concussion symptoms, with the OFC lesion group reporting more overall symptoms at the moment (OFC = 16.75 ± 15.14 points vs. Controls = 2.54 ± 5.52 points, *p* = 0.005). The OFC lesion group reported more somatic and cognitive symptoms, but the difference in emotional symptoms between the groups did not reach significance. The scores, SD, and *p*-values are listed in Table 3.

**Table 3:** Results of the questionnaires.

		Orbitofrontal		Control		
Questionnaire	Scale	Mean	SD	Mean	SD	*p* value
BRIEF—A	Global Executive Composite	61.3	13.6	49.2	9.7	0.026*
	Behavioral Regulation Index	58.7	15.3	50.5	9.6	0.240
	Metacognition Index	62.0	12.1	48.6	9.2	0.007*
	Inhibit	55.5	10.3	47.7	8.5	0.013*
	Shift	56.4	9.7	48.7	9.1	0.039*
	Emotional Control	58.3	16.8	52.1	9.8	0.639
	Self-Monitor	57.6	16.2	49.9	11.9	0.217
	Initiate	60.1	11.5	48.8	7.5	0.015*
	Working Memory	65.1	16.5	48.5	10.6	0.005*
	Plan/Organize	59.6	9.7	48.2	8.2	0.007*
	Task-Monitor	63.1	14.1	51.3	8.6	0.075
	Organization of Materials	55.7	9.6	48.9	9.3	0.082
RPQ	Total at the moment	16.8	15.1	2.5	5.5	0.005*
	Somatic symptoms	7.0	7.75	1.0	2.03	0.007*
	Emotional symptoms	3.5	3.9	0.7	2.3	0.059
	Cognitive symptoms	5.0	3.8	0.6	2.0	0.0005*
BDI		6.7	5.4	2.9	3.4	0.080

*Mean scores and standard deviations (SD) for the Behavior Rating Inventory of Executive Functions—Adult version (BRIEF-A), Rivermead Post-Concussion Symptoms Questionnaire (RPQ) and Beck’s Depression Inventory (BDI) for both groups are presented. The *p*-values are acquired from between-group comparisons for each category. Statistical significance is highlighted with bold values and an asterisk*.

## Discussion

In this study, we found evidence for altered emotional modulation of attention and cognitive control related brain responses in patients with OFC injury. In contrast to healthy subjects, patients with OFC lesion did not initially allocate additional attentional resources in Go-situation when faced with threat-related distractors as evidenced by unchanged N2P3 amplitude. Instead, they showed delayed and prolonged processing of emotion, seen as enhanced late positivity during LPP. Furthermore, these patients showed increased early allocation of cognitive control resources in NoGo-situation in the context of emotional distractors, reflected in increased N2 potential. In summary, OFC lesion resulted in altered impact of task-irrelevant threat-related distractors on task-related attentional and cognitive control processes. Altered emotional modulation of ongoing attentional and executive processes, such as delayed and prolonged impact of emotion and increased allocation of cognitive control resources when task-related cognitive control demands and emotion-related cognitive control demands meet, may underlie some of the challenges patients with OFC lesion encounter in everyday life.

The role of the OFC in attention remains to be established even though several of its presumed functions are closely related to attentional processes. While the OFC is not directly associated with spatial attention (Kennerley and Wallis, 2009), it contributes to attentional choices by representing reward value, calculating potential outcomes and in redirecting value information to other executive control related brain areas (Rolls, 2004; Wallis, 2007), thus guiding where attention should be directed. The OFC, along with ACC and temporo-parietal region, has been suggested to be part of the neural system responsible for monitoring potentially behaviorally relevant stimuli even outside the focus of attention (Gruber et al., 2010). In the current study, we found evidence for altered interplay in this network due to lesion to OFC. Patients with OFC lesion failed to show the typical impact of emotional distractors in attention-related ERP components observed in the Control group on electrodes over the right frontal, central and parietal regions. While no source analysis was conducted it is likely that attention-related ERP components modulated by emotional stimuli at frontal, central and parietal electrodes depict activity in this aforementioned neural system comprising of OFC, ACC and temporo-parietal brain regions. Thus, we speculate that lesion to OFC, a crucial part of this network, consequently results in altered interplay even in the intact brain regions that are part of this neural system responsible for monitoring potentially behaviorally relevant stimuli including emotional stimuli. fMRI studies have found the OFC to be activated even when processing random, non-emotional figures which were not in the focus of attention but relevant for the task (Diekhof et al., 2009) and activated in a similar manner when processing emotional content or encountering any salient, behaviorally relevant stimuli with no affective value (Diekhof et al., 2011). Thus, the OFC links information about the salience and emotional and motivational aspects of stimuli to brain areas involved in ongoing tasks influencing attentional and cognitive processing. A lesion to the OFC and to neurons connecting it to other regulatory brain areas may alter the balance in attention allocation to emotional stimuli as shown in our current and previous studies (Hartikainen et al., 2012a; Mäki-Marttunen et al., 2017).

We have previously reported that a lesion to the OFC alters the balance in attention allocation between emotional and non-emotional targets so that patients with OFC lesion allocate even more attentional resources to emotional targets than healthy control subjects (Mäki-Marttunen et al., 2017). OFC lesion leading to prioritizing targets over distractors to a greater extent than healthy subjects, has also been reported in studies using non-emotional targets (Hartikainen et al., 2012a; Løvstad et al., 2012b). Similarly, in the current study the OFC group’s attention allocation in the Go-situation was prioritized to task-relevant, non-emotional targets and initially no additional attentional resources were allocated due to threat-related distractors, unlike in the Control group. We speculate that after task completion (i.e., motor response), sufficient attentional resources were released allowing processing of the emotional distractor.

We further speculate that besides the stronger than normal bias of attention to targets over distractors, the early emotion-related activation and late regulation of emotion were impaired due to OFC lesions, leading to problems in initial attention allocation to task-irrelevant threat and prolonged duration of it later. The medial and lateral OFC have been assigned distinct roles with the lateral OFC linked to suppression or inhibition of emotional information (Hooker and Knight, 2006) and the medial OFC linked to identifying and monitoring emotion-related value (Kringelbach and Rolls, 2004; Hooker and Knight, 2006). Our group of patients included subjects where both lateral and medial OFC were lesioned. Negative emotion has been suggested to initiate early and strong activation of the medial OFC (Northoff et al., 2000). Task-irrelevant but biologically relevant emotion needs to be attended to swiftly in order to evaluate whether it requires instant actions that would overrun the task at hand. Delayed attention to threat may predispose one to danger and to challenges in everyday situations such as social interactions where socially relevant emotional cues irrelevant to current personal goals should be attended to immediately for good interaction because they include important emotional content. Successful social and goal-related behaviors, on the other hand, require ability to suppress emotional reactions when uncalled for.

As expected, increased N2P3 amplitude towards threatening emotional distractors was detected in healthy controls in Go-situation. The enhancement of N2P3 amplitude was observed especially over the right hemisphere, consistent with right hemisphere dominance in emotional processing and emotion-attention interaction (Hartikainen et al., 2000, 2007, 2010a; Demaree et al., 2005). The right-lateralized emotion-attention interaction in healthy subjects is supported by negative emotional stimuli interfering with right hemisphere dependent processes, such as detection of left visual field targets (Hartikainen et al., 2000, 2007) and global as opposed to local visual feature processing (Hartikainen et al., 2010a). Behavioral interference due to emotional stimuli and task-relevant stimuli competing for the right-hemispheric processing resources is also reflected in attention-related ERPs over the right hemisphere (Hartikainen et al., 2007, 2010a). The right-lateralized enhancement of N2P3 amplitude in the context of emotional distractors in the current study is in line with previous studies reporting enhanced attention to emotion (Schupp et al., 2007; Hajcak and Olvet, 2008; Carretié, 2014) and novelty in healthy subjects (Daffner et al., 2000). Moreover, in the current study the Control group showed no threat-related increase in amplitudes after the P3 potential, i.e., the LPP, indicating that the effect of task-irrelevant threat on task processing was successfully regulated (Hajcak et al., 2009).

In contrast to the Control group, patients with lesion to the OFC did not show increased attention to task-irrelevant threat at the N2P3 amplitude. Instead, they had increased late positivity (LPP) in the context of the emotional distractor which was lateralized to the left parietal region. Increased LPP has been reported in previous studies where healthy subjects viewed emotional content (Hajcak et al., 2009). The LPP is suggested to reflect motivated attention (Ferrari et al., 2008), emotion regulation during reappraisal tasks (Hajcak and Nieuwenhuis, 2006) and inhibition of automatic emotional processing (Diedrich et al., 1997). Successful reappraisal of emotional content reduces the LPP compared to a situation when emotion is only attended to (Hajcak and Nieuwenhuis 2006). It is possible that the OFC lesion group failed in the timely evaluation of the significance of threat and in subsequent inhibition or control of attention to emotional distractors, leading to pronounced LPP. The observed effect of emotion lateralized to the left parietal region in patients with OFC lesion, as opposed to typical lateralization to right hemisphere in healthy subjects, is in line with previous literature showing asymmetrically enhanced ERP patterns after bilateral orbitofrontal damage thereby suggesting OFC having distinct modulatory effects of left and right hemispheres (Hartikainen and Knight, 2003). We suggest that the observed alterations in time course of emotional modulation of attention may underlie some of the real-life challenges patients with OFC lesion encounter with delayed impact of emotion, potentially having downstream consequences influencing the impact of emotional events on behavior.

In addition to altered dynamics of emotion-attention interaction observed in Go-trials, altered dynamics of emotion and cognitive control was observed in NoGo-trials in the OFC lesion group. In NoGo-trials, both groups showed increased N2P3 amplitudes towards the emotional distractor, but further analysis in time windows 600–700 ms and 700–800 ms and on separate N2 and P3 peaks revealed differences in the temporal dynamics of brain responses to threat. Patients with OFC lesion showed increased N2 amplitudes in the context of threat, whereas in Control group threat-related increase in brain potentials was located at P3. The N2 is suggested to reflect early cognitive control and conflict monitoring in a NoGo response inhibition task, whereas the NoGo P3 is suggested to reflect later phases of response inhibition and cancellation of the planned response (Groom and Cragg, 2015). Thus, OFC lesion may shift the phase where threat-related emotional stimuli interact with cognitive control processes needed for response inhibition from later P3 to earlier N2. Response inhibition performance, i.e., the amount of Commission errors, didn’t differ between the groups and the amount of Commission errors was not influenced by emotional valence of the distractors. Thus, the changed emotional modulation of cognitive control did not affect task performance and furthermore, no objective response inhibition deficiency was detected.

Based on the results of the NoGo-condition, we suggest that patients with OFC lesions allocated more cognitive control in the early phase of the response inhibition task controlling for the possible distraction generated by threat, thus achieving similar task performance as healthy subjects. In Control group, good performance was seen without increased early cognitive control and the impact of threat was shown in later attention or response cancellation related P3 potential. We reported increased allocation of early cognitive control resources in the context of task-relevant threat-related stimuli in patients with OFC lesion, evidenced by increased N2 in NoGo-trials, in our previous study (Mäki-Marttunen et al., 2017). In that study task performance was relatively improved in the context of task-relevant threat. To that end, the current results suggest that task-irrelevant emotional stimuli may require more intense cognitive control to maintain task performance when cognitive control demands of task-irrelevant threat meet with the cognitive control demands related to the task itself.

While structured and emotionally neutral neuropsychological testing environments fail to provide objective evidence for reported subjective challenges in every-day executive functions in patients with OFC lesion, reported real-life challenges may well be related to situations where several executive functions are engaged simultaneously and emotional events meet with cognitive demands. In real-life situations, emotional information is always present in social interactions and decision-making, thus executive functions are rarely performed in an emotionally neutral environment. In the current study we found both objective and subjective evidence for mild executive dysfunction in the OFC group. The OFC lesion group committed more errors in general and missed responding more frequently in the Executive RT Test and reported more challenges with executive functions in the BRIEF-A questionnaire compared to the Control group. While the errors were not directly related to the emotional dimension of the distractor we speculate that the impaired performance observed in patients with OFC lesion may be partly due to difficulties in balancing attentional and executive function resources in a task with distracting emotional stimuli. Furthermore, prolonged processing of emotional distractors may hinder readiness to attend to the upcoming target on the following trial, predisposing one to errors. We suggest the impaired task performance, combined with delayed attention allocation to threat, reflects disruption of the executive-emotional control networks due to lesion to the OFC and the core difficulties lie in balancing attention to task-relevant non-emotional information vs. task-irrelevant but biologically or socially relevant emotional information.

There are few limitations to the current study. The small sample size limits statistical power and predisposes to false negative findings due to lack of power and false positive findings by chance due to differences between the groups which are not related to the brain lesion. Thus, more reliable results could have been obtained with more participants. However, it is very difficult to find a large cohort of patients with small lesions restricted to the OFC and multiple previous studies have used similar sample size as used in this study (Paulmann et al., 2010; Funderud et al., 2013; Mäki-Marttunen et al., 2017). Lesion studies where certain brain functions are compared in a group of subjects with focal brain lesion to those with intact brain circuits is a traditional and powerful method to study brain structure–function relationship and invaluable information has been obtained with only a few subjects (Turken and Swick, 1999). Thus, we believe our sample size is acceptable and provides trustworthy results. In the current study subject’s lesion sizes varied and patients with both unilateral and bilateral lesions were included. This may limit conclusions related to how specific anatomical regions of the OFC contribute to the findings of this study. Additionally, some patients had lesions in other frontal regulatory brain areas which could confound the results. Finally, unequal distribution of males and females in the OFC lesion group and the Control group is a potential confounding factor. However, the reported results are unlikely to be explained by gender differences and we are not aware of any literature suggesting such differences in emotion-attention interaction due to gender.

While there are weaknesses there are also important strengths to the current study. Most of the current knowledge on the role of OFC stems from animal studies or human imaging studies (Kringelbach and Rolls, 2004; Wallis, 2012; Rudebeck and Murray, 2014). Translating knowledge from animal studies to humans, especially regarding emotion guided behaviors, has its limitations related to methodological issues and obvious differences in brain and behavior between species. fMRI lacks the temporal resolution of EEG which is needed for studying millisecond level temporal dynamics of mental events. Furthermore, fMRI is not well-suited for studying the OFC, as it is located next to air-filled sinuses predisposing to susceptibility artifacts i.e., distortions or local fMRI signal change due to local magnetic field inhomogeneities (Kringelbach and Rolls, 2004). In addition, OFC activations observed in fMRI studies do not provide causal information on the role of OFC in a given task. There are currently only a handful of electrophysiological studies on patients with focal brain lesion to OFC which allow for unique insight into the role and neural dynamics of human OFC. Thus, while access to the number of subjects with focal OFC lesion is limited, the current study and other similar studies provide a powerful means to better understand the role of OFC in human emotion, cognition and behavior.

## Conclusion

We detected altered processing of task-irrelevant emotional distractors in patients with OFC lesion. In Go-situation, the OFC lesion group did not show early increase in attention allocation to threat-related distractors whereas a late positivity in the context of emotional distractors was detected. Control group showed increased attention to task-irrelevant threat, seen as increased N2P3 amplitude on the right hemisphere. Performance of the OFC lesion group was slightly compromised in the executive function task evidenced by an increased amount of Total errors and Missed responses. In NoGo-condition, the OFC lesion group performed equally to the Control group and they had similarly enhanced N2P3 amplitude in the context of threatening distractors. However, the time course of emotion-related enhancement in attention differed between the groups so that OFC group showed increased N2 potential, i.e., increased cognitive control, compared to larger P3-potentials in the Control group. The OFC lesion group reported more cognitive symptoms and challenges with executive functions than the Control group but no impairment in emotional control in subjective measures.

We conclude that lesion to the OFC alters neural dynamics underlying attention allocation to task-irrelevant emotion. We suggest that lesion to the OFC impairs individual’s ability to balance attention between task-relevant targets and task-irrelevant distractors and changes the underlying neural processing by delaying and prolonging processing of task-irrelevant emotional stimuli. On the other hand, successful execution of response inhibition may require additional neural resources at early phases of the cognitive control process, especially in emotional contexts. The interaction of attentional, executive function and emotional processing is compromised, leading to challenges in integrating emotional stimuli into current goals and actions. Combining tests assessing emotion, attention and executive function interaction, such as the Executive RT Test, along with the ability to detect alterations in dynamics of underlying brain processes with ERPs, may be beneficial in assessing the changed neural circuits and dynamics after OFC lesion and in understanding the mechanisms behind the every-day challenges these patients encounter.

## Data Availability

Restrictions apply to the datasets. The datasets for this manuscript are not publicly available because the written consent forms signed by the participants did not provide information concerning public distribution of data.

## Author Contributions

VK had the main responsibility of data collection, data analysis and writing of the manuscript. EC was also responsible for data collection, initial analysis and helped with the writing process. JP contributed to data analysis and the writing process. KO and KH contributed to writing and brought important intellectual content to the article. KH was responsible for the whole process and supervision of this research project. All authors contributed to reading and revising the manuscript and approved the submitted version of it.

## Conflict of Interest Statement

The authors declare that the research was conducted in the absence of any commercial or financial relationships that could be construed as a potential conflict of interest.
